# Comparing Face-to-Face, Blended and Online Teaching Approaches for Practical Skill Acquisition: A Randomised Controlled Trial

**DOI:** 10.1007/s40670-024-02026-8

**Published:** 2024-04-04

**Authors:** Cuisle Forde, Annie O’Brien, Ovidiu Croitoru, Nadine Molloy, Chiara Amisano, Iain Brennan, Adam McInerney

**Affiliations:** 1 Discipline of Physiotherapy, School of Medicine, Trinity College Dublin, Dublin, Ireland; 2 School of Medicine, Trinity College Dublin, Dublin, Ireland; 3 Department of Physiology, School of Medicine, Trinity College Dublin, Dublin, Ireland

**Keywords:** Clinical skill, Online education, Practical education, Student voice

## Abstract

**Introduction:**

The efficacy of blended and online teaching methods for practical skill acquisition remains ambiguous, particularly for skills requiring haptic awareness and/or sensory training. This study aims to compare three teaching methods (face-to-face, blended, online) for the acquisition of skills requiring sensory learning and haptic awareness. A secondary aim was to explore student experience of each teaching approach.

**Design:**

A post-test only randomised controlled trial.

**Methods:**

Forty-seven participants chose between learning two skills: manual measurement of blood pressure during exercise (BPM) and/or skin fold measurement using callipers (SKM). Participants were randomised to one of three learning groups: face-to-face (*n* = 23), blended (*n* = 22) and online (*n* = 26). Assessors determined skill competency during an in-person skill demonstration session. A survey captured student experiences.

**Results:**

For SKM, there was a statistically significant difference in skill competency between the online learning group (17% achieved competency) and both the face-to-face (75% achieved competency; *p* = 0.011) and blended (89% achieved competency; *p* = 0.001) learning groups. For BPM, the online group had the lowest percentage of participants achieve overall skill competency. Both knowledge-based and sensory-based sub-competencies were negatively affected by the online learning method. For both skills, students in the face-to-face and blended learning group were significantly more confident in their knowledge and their ability to perform the skill in a clinical setting, compared to the online learning group.

**Conclusion:**

Both face-to-face and blended teaching methods were more effective at leading to skill acquisition and were preferred by students when compared to a fully online teaching method.

## Introduction

Technology has facilitated rapid advancements in medical education [1]. It has had a transformative impact since the onset of the Covid-19 pandemic, taking a central role in the future of teaching and learning [2]. Recent years has seen technology increasingly integrated into pedagogical practices to support both theoretical learning and skill acquisition in the form of blended or hybrid learning. In fact, courses that are not supported by a virtual learning environment and/or learning management system in some form or another are hard to come by, and students use both official and non-official technologies to support their learning [3, 4].

Clinical skill teaching is an essential component of health science education and an area many would not traditionally consider suited to remote learning. However, technology played a pivotal role in supporting distant teaching of both hard and soft skills during the Covid-19 pandemic [4]. PowerPoint presentations supported remote anaesthesia training [5], online skill demonstrations supported suturing competency [6] and web-based simulations supported patient assessment and diagnosis [7].

Digital medical education will continue to evolve as educators change how they teach practical skills, yet there is ambiguity around the role of technology in supporting or impeding practical skill acquisition. Some studies have found face-to-face teaching superior to online learning approaches [8, 9], while others have identified blended learning as effective in building practical skill competency [6, 9–12]. There is however a dearth of literature comparing the efficacy of all three teaching methods (i.e., face-to-face, blended and online) in practical skill acquisition. There is also ambiguity surrounding the skills, or aspects of these skills, that can be successfully taught using online or blended teaching approaches [13]. Meta-analyses and qualitative research has found that technology successfully supports the acquisition of low-stake skills [9, 14]. Skills requiring haptic awareness, manual dexterity and auditory training may however require in-person practice and feedback to consolidate [15]. A systematic review has determined that many studies of digital medical education are low-quality [16]; reviews predominantly compare digitally enhanced teaching against one other teaching approach, i.e. traditional or digitally enhanced teaching [1], and many examine theoretical skill learning rather than practical skill acquisition. Furthermore, many studies measure skill acquisition using self-report measures [17, 18] which are less reliable for knowledge assessment among healthcare students compared to objective measures [19].

A randomised controlled trial exploring the effectiveness of three teaching methods in knowledge and skill acquisition of anaesthesia application identified no significant difference between groups [5]. Scored out of 180, the in-person (x̄ = 161), blended (x̄ = 154) and online (x̄ = 152) learning groups achieved high mean competency scores across groups. While this skill requires technical fluency, soft skills (e.g. patient handling, communication) are an essential and core component of anaesthesia application [20]. It is therefore possible that the 3rd year students participating in this study had already practiced and achieved a baseline competency in these soft skills by their 3rd year. Unfortunately, this study did not include data pertaining to the sub-competency achievements and whether students achieved the more manual sub-competencies compared to the soft skill sub-competencies. Another study exploring in-person and online approaches to learning electrocardiogram electrode placement found the groups comparable across all skill sub-competencies bar electrode placement, whereby online learners performed significantly worse [21]. Greater understanding of the suitability of skills requiring sensory training is therefore needed to enhance understanding of the types of skills necessitating in-person practice.

It is important to identify the most effective pedagogical approaches to practical skills teaching to advance practical skill teaching moving forwards [13, 21]. Identifying the components of a skill that are successfully acquired across different teaching modalities will enable educators to make evidence-informed decisions when considering methods of skills teaching [13, 22]. The creative and innovative techniques employed by educators during the pivot to distant learning will undoubtedly continue to support practical skill teaching moving forwards post-pandemic [2]. However, to maximise learnings from the pandemic, we must address a key gap in knowledge, namely, whether there is a superior teaching method (face-to-face, blended, online) to support practical skill acquisition in health science students. Studies comparing multiple different methods can generate more meaningful and generalisable findings [1]. It is also important to recognise that while there are skills that may not be suited to distant learning, there may be skill components that can successfully be acquired using digitally enhanced teaching approaches. Clinical skills are comprised of individual subskills or sub-competencies that are necessary to carrying out an overall skill effectively and safely [23]. Identifying the components of a skill suited to digitally enhanced teaching can build an evidence-base for educators and inform their approaches to future practical skill teaching.

The primary aim of this study was to compare three teaching methods through examining overall competency achievement and sub-competency achievement for two practical clinical skills. A secondary aim was to evaluate student satisfaction of these teaching methods and student confidence in their ability after teaching has been completed. Two skills were chosen for this study: calculating body fat percentage using skinfold measurement (SKM) and manually measuring blood pressure during exercise (BPM). These two skills were chosen as they are skills that require sensory training or a degree of haptic awareness. Tactile training and manual dexterity are required in the case of SKM, and auscultation and tactile training are required for BPM. Furthermore, while they are clinically relevant [24, 25], undergraduate health science students are likely to be naïve to the manual measurement of these skills. It was hypothesised that students in the fully online group would not perform as well as students in the other two groups and that differences would be seen between groups in sub-competencies related to sensory learning rather than knowledge.

## Materials and Methods

The CONSORT checklist [26] was followed to ensure quality reporting of this trial and can be found on the Open Science Framework (OSF; https://osf.io/3pezg).

This study employed a cross-sectional parallel design using a 1:1 allocation ratio, with participants randomised into one of three learning groups: a face-to-face learning group, a blended learning group and an online learning group. Participants were offered the choice of learning one or both of the practical skills being taught: manual measurement of blood pressure during exercise (BPM) using a sphygmomanometer, or calculating body fat percentage using skinfold measurement (SKM) with callipers.

### Participants

Undergraduate students at Trinity College Dublin’s School of Medicine were invited to take part. Class representatives of each undergraduate class were asked via email to circulate a recruitment poster among their class inviting students to participate in the study. Students were eligible to take part if they were (i) over the age of 18; (ii) naïve to the skill being taught; and (iii) undergraduate students of Trinity College Dublin’s School of Medicine. Inclusion criteria were based upon a requirement for participants to have a basic knowledge of anatomy and physiology to acquire the relevant skills. Participants were not eligible to take part if they were under the age of 18, had previously received training in their chosen skill or were not enrolled in a course within Trinity College’s School of Medicine.

### Procedure

This research benefited from public and patient involvement (PPI) in the form of a student advisory group (SAG). The SAG comprised of 12 1st-3rd-year health science student volunteers, from a range of health science disciplines (e.g. medicine, nursing, pharmacy). Consistent with our methodological approach, the SAG was involved at all stages of this work in an advisory capacity. Their feedback was used to inform and consolidate the authors’ work as the project progressed. Teaching materials for both skills were designed, developed and piloted by members of the research team prior to participant recruitment, which began in January 2022, and minor amendments were made to the teaching materials accordingly. The trial was carried out between February and March 2022. Those who expressed interest in taking part were emailed a participant information sheet and a consent form, and indicated which skill they would like to learn. Upon receipt of written informed consent, students were randomised to the face-to-face, blended or online learning group for their chosen skill(s) via the procedure of simple randomisation using a random number generator powered by Google. NM generated the random allocation sequence, informed AOB of each participant’s group allocation and AOB enrolled and assigned participants to groups. An email was sent to each student informing them of their learning group allocation, their unique participant identification code and a request to indicate their availability for the in-person demonstration. Participants randomised to the face-to-face and blended teaching groups were also asked to indicate their availability for in-person teaching sessions. All participants were provided with the necessary equipment to practice their chosen skill in their own time free of charge. Students assigned to BPM received a sphygmomanometer and stethoscope and students learning SKM received skinfold callipers. In-person teaching took place in the exercise laboratory on the ground floor of Trinity’s Centre for Health Science at St. James’ Hospital, Dublin. Teaching sessions had a minimum of 4 and maximum of 10 students and were taught by the principal investigator of the project.

All participants were enrolled onto the online learning management system Blackboard; however, access to specific sections of the relevant module was restricted based on teaching group assignment (see Table 1). Three hours of student engagement per skill was required across all learning groups. Material covered in-person was as similar as possible to that provided online for other groups (see Table 2). For example, the same slides were used to develop the online interactive presentation as were used in the in-person presentation, and the same content was covered and taught by the same person such that only the teaching modality differed between groups. Students could access the online material as many times as they wanted. Where the face-to-face and blended groups received a practical class to practice the relevant skill, the online group was provided with a detailed demonstration video, encouraged to practice the skill on family and/or friends in their own time and asked to complete and submit an online data sheet detailing the results of their practice.

**Table 1:** Access to online teaching materials across learning groups

**Online module sections**	**Section information**	**Face-to-face**	**Blended**	**Online**
Introduction	Details of the learning objectives	Yes	Yes	Yes
Prepare	Interactive website providing information about the skill	No	Yes	Yes
Study-theory	Pre-recorded video of the instructor detailing the theory of the skill (e.g. definition and importance, contraindications, steps to successfully complete the skill and common mistakes made)	No	Yes	Yes
Study-videos	Pre-recorded video of the instructor demonstrating the skill with a volunteer	No	No	Yes
Apply	Encouraged participants to practice their skill at home and share their results through an online form	No	No	Yes
Reflect	An academic paper detailing mistakes to avoid when carrying out the skill to improve accuracy	No	No	Yes
Recall	A multiple-choice questionnaire to test knowledge of skill theory	No	Yes	Yes
Extend	Three additional academic papers to expand knowledge	Yes	Yes	Yes

**Table 2:** Access to in-person teaching by learning groups

**In-person teaching**	**Detailed information**	**Face-to-face**	**Blended**	**Online**
Theory class	In-person class where the instructor provides information about the skill and theory of the skill (e.g. definition and importance, contraindications, steps to successfully complete the skill and common mistakes made)	Yes	No	No
Practical class	Instructor demonstrating the skill with a volunteer; participants given the opportunity to practice the skill; individual feedback from the instructor including how to avoid mistakes etc	Yes	Yes	No
**Competency demonstration**	All participants asked to demonstrate their chosen skill and complete a questionnaire	Yes	Yes	Yes

### Details on Teaching Methods

The face-to-face learning approach consisted of a single in-person teaching session with an instructor lasting 2 h. The first hour was spent with an instructor teaching skill theory. The second hour was an in-person practice session with the instructor teaching participants how to carry out the skill on a member of the research team (i.e. a demonstrator), with time allocated for participants to practice the skill on demonstrators and volunteers. Participants had the opportunity to ask questions and receive feedback from the instructor.

The blended learning group engaged with online teaching materials prior to an in-person teaching session. The online learning materials consisted of self-directed study including pre-recorded videos of the instructor teaching skill theory. This was followed by a 1-h in-person practice session with an instructor who demonstrated and performed the skill. Students had time to practice the skill with volunteers, ask questions and receive feedback on their technique. This group also had access to a short multiple-choice quiz online.

The online learning group had access to interactive presentations, detailed demonstration videos of the relevant skill and a moderated online discussion forum with tasks to complete. Task necessitated practicing the skill in their own time. They were also provided with a multiple-choice quiz online. Participants in the online group did not attend a practical in-person class.

All participants had access to their learning objectives and three additional academic papers relevant to the skill they were learning (analogous to a reading list) in the “extend” section through the learning management system.

### Competency Demonstration

To determine if skill competency was achieved, all participants attended an in-person competency assessment 2 weeks after teaching was completed. Participants were assessed on their technique, accuracy and their professionalism across the skill sub-competencies. Skill gross and sub-competencies are detailed in Table 3. Each sub-competency was scored dichotomously (achieved/not achieved) and overall skill competency was determined by whether the participant obtained the correct blood pressure or body fat percentage. As the primary investigator was both the instructor and assessor, a blinded assessor also assessed the first 25% of the demonstrations. This was done in person in real time with both assessors being present as students were completing the relevant skill. The blinded assessor did not know which teaching group participants had been allocated to. Where there was a discrepancy in scoring between assessors, the assessors conferred until an agreement was met. After approximately 20% of participants were assessed, agreement between assessors was consistently very high, regardless of the skill being demonstrated. It was therefore considered unnecessary to have two assessors present during further demonstrations. After demonstrating skill competency, participants completed a survey to capture perceptions of the learning experience and confidence in their knowledge, demonstration and performance of the skill, using a 5-point Likert scale. The survey was developed by the research team, piloted by medical students, and is available through the Open Science Framework (OSF; https://osf.io/y7tuj).

**Table 3:** Assessment criteria for overall skill competency and skill sub-competencies

**Sub-competency**	**Skill**	
	**BPM**	**SKM**
**1**	Correct patient position	Skinfold sites known
**2**	*Accurate position of cuff	*Skinfold sites accurately located
**3**	*Correctly applied cuff	*Callipers positioned correctly
**4**	*Stethoscope positioned correctly	Callipers read correctly
**5**	*Patient’s arm positioned correctly	*Callipers released before skinfold
**6**	Patient’s arm is supported	Rotation of sites
**7**	Quiet observed	Minimum of 2 measurements per site
**8**	Adequately inflated cuff	*Accurate skinfold measurements
**9**	*Pressure released adequately	Calculation of predicted body density achieved
**10**	Measurement repeated after 30 s rest	Calculation of body fat % achieved
**Overall competency**	Correct blood pressure reading obtained	Correct body fat % obtained

*BPM* refers to manually measuring blood pressure during exercise, *SKM* refers to calculating body fat percentage using skinfold measurement

*A sub-competency which requires a degree of sensory learning (i.e. palpation, haptic awareness etc.)

### Data Analysis

Data was analysed using IBM SPSS version 26 (IBM Corporation, Armonk, NY, USA). Survey data was tested for normality using Shapiro–Wilk tests, where *p* < 0.05 was considered statistically significant; Kruskal–Wallis non-parametric tests were used as the data was not normally distributed. Mann–Whitney post hoc tests were carried out with a Bonferroni correction. To evaluate the total amount of sub-competencies achieved by a participant during the competency assessment, each sub-competency was dichotomously coded; an overall sub-competency achievement score was calculated by adding each point and calculating an overall mean score. For survey results, Fisher’s exact test was used to determine differences in responses between teaching method groups.

## Results

**Fig. 1:**
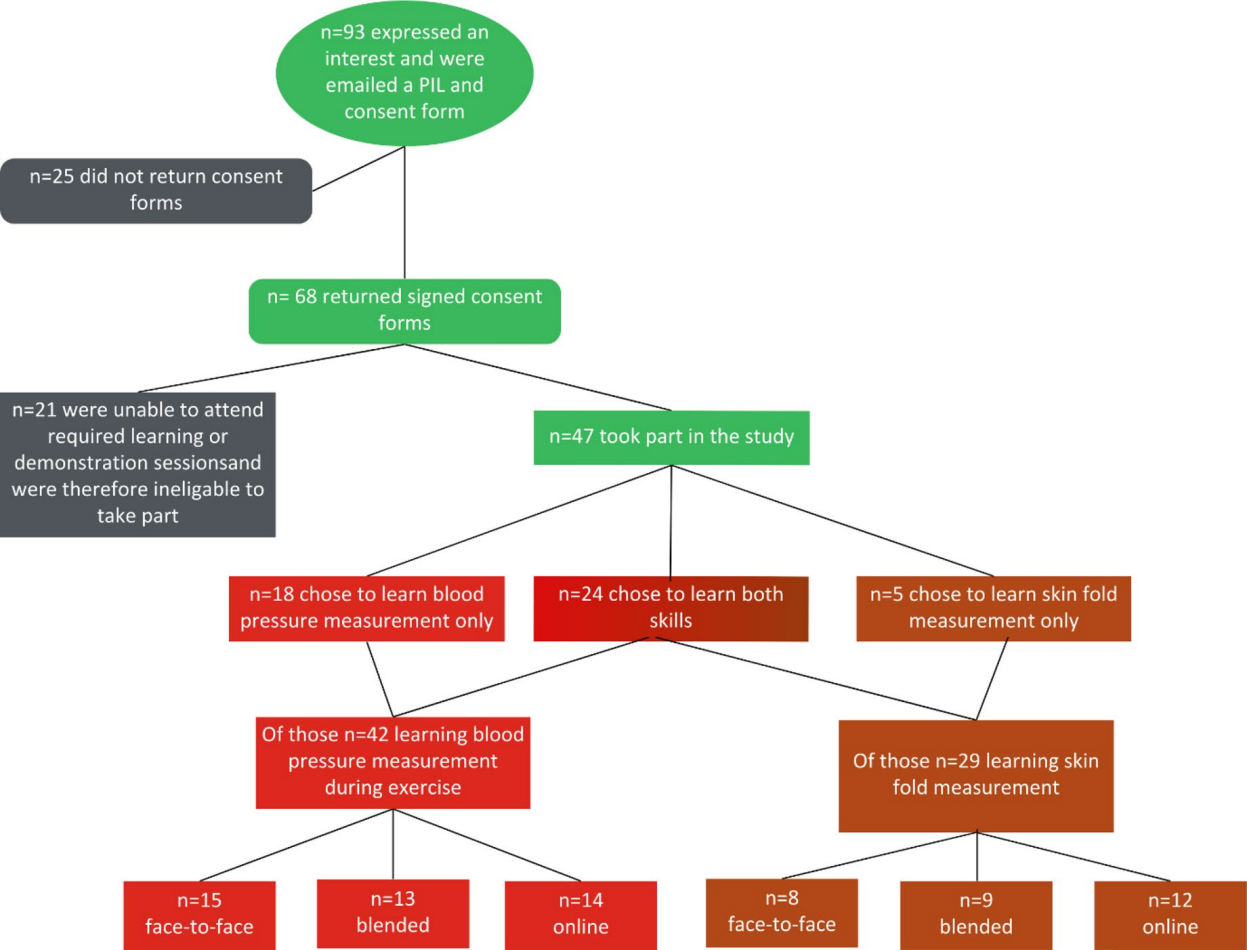
Participation and group allocation indicating number of participants in each teaching method group and number of participants learning each skill

Of the 93 students who expressed an interest in taking part, 68 returned signed consent forms. During email correspondence with potential participants regarding availability to attend in-person teaching sessions and competency assessments, a further 21 students did not confirm their attendance and were therefore not eligible to take part in the study. Overall, 47 students took part in the in-person skill demonstration indicating an attrition rate of 31% across the course of the study. Figure 1 outlines the numbers of students who learnt each skill. Of the participants learning manual blood pressure measurement during exercise (BPM; *n* = 42), 15 were in the face-to-face group, 13 in the blended group and 14 in the online group. Of the participants learning how to calculate body fat percentage using callipers (SKM; *n* = 29), 8 were in the face-to-face group, 9 in the blended group and 12 in the online group.

### Overall Skill Competency

**Fig. 2:**
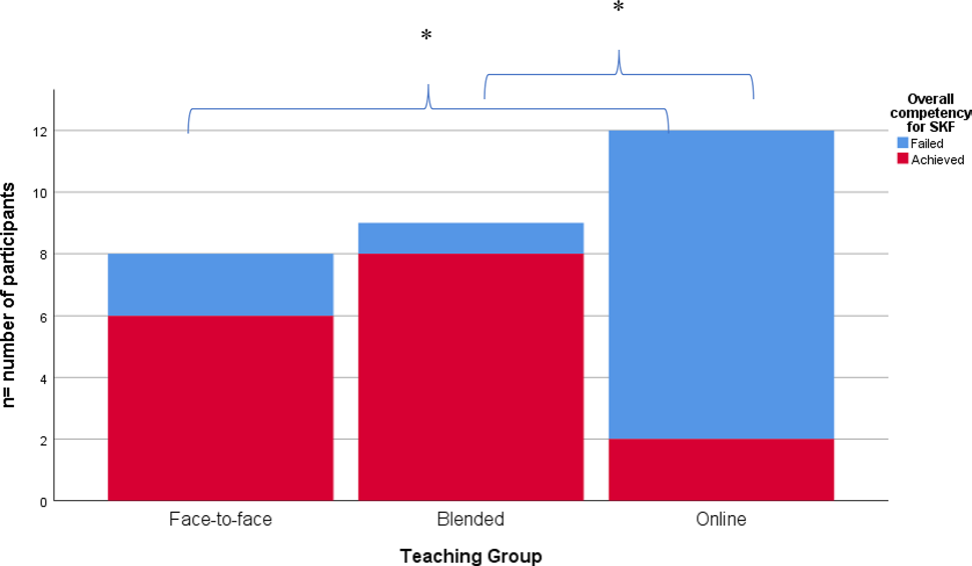
Overall achievement of calculating body fat percentage using skinfold measurement (SKM) by learning group. Note: * indicates a statistically significant difference between learning groups

A Kruskal–Wallis test was performed on overall competency between groups (face-to-face, blended and online) for both BPM and SKM. For SKM, the differences between the mean rank score of 17.88 (face-to-face), 19.89 (blended) and 9.42 (online) were statistically significant, *H*(2,*n* = 29) = 12.168, *p* = 0.002. Post hoc tests revealed a statistically significant difference in student overall competency of SKM between the online learning group (17% achieved competency) and both the face-to-face (75% achieved competency, *p* = 0.035) and blended (89% achieved competency, *p* = 0.004) learning groups. There was no significant difference between face-to-face and blended learning groups (see Fig. 2).

**Fig. 3:**
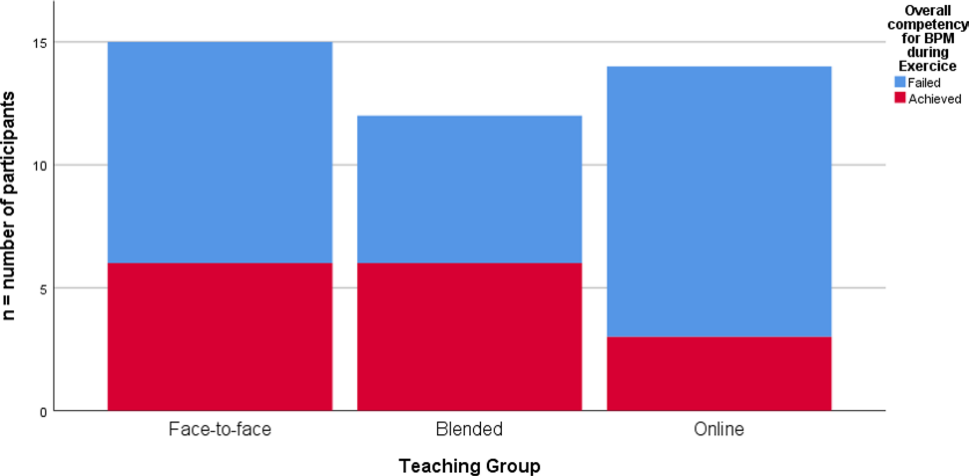
Overall achievement of manually measuring blood pressure during exercise (BPM) by learning group. No significant between-group differences were detected

For BPM, the online group also had the lowest rate of overall competency achievement; however, there was no statistically significant difference found across the face-to-face (40% achieved competency), blended (46.15% achieved competency) or online (21.42% achieved competency) learning groups (see Fig. 3).

### Skill Sub-competency

A Kruskal–Wallis test was performed on the total sub-competency scores of the three groups (face-to-face, blended and online) for both BPM and SKM. For BPM sub-competency, the difference between the mean rank score of 24.27 (face-to-face), 25.50 (blended) and 14.82 (online) was statistically significant, *H*(2, *n* = 42) = 6.52, *p* = 0.038. Between-group differences could not be detected by post hoc tests with a Bonferroni adjusted alpha level. When individual sub-competencies were examined, a statistically significant difference was seen between groups (*p* = 0.019) for correct cuff placement, with a higher rate of failure among participants in the online group.

For SKM sub-competency, the difference between the mean rank score of 19.81 (face-to-face), 19.11 (blended) and 8.71 (online) was significant, *H*(2, *n* = 29) = 12.71, *p* = 0.002. Between-group differences were detected by post hoc tests with a Bonferroni adjusted alpha level. The difference between face-to-face and online groups was statistically significant (11.10, *p* = 0.007) and the difference between blended and online groups was also statistically significant (10.40, *p* = 0.010) but there was no difference between blended and face-to-face groups (0.70, *p* = 1). When individual sub-competencies were considered, it was noted that those in the online group were significantly more likely than those in the other groups to fail three specific sub-competencies: repeating measurements (*p* = 0.042), rotating sites (*p* = 0.042) and accurately reading the callipers (*p* = 0.032).

### Student Experiences

Of the 42 participants surveyed, 31 (74%) agreed that digital technology had a place in teaching practical skills, 4 participants (9.5%) felt that there was no place for digital technology in teaching practical skills and the remaining 7 participants were unsure (16.5%). When asked their preferred teaching method for practical skill teaching, no participant chose online learning; the majority expressed a preference for blended learning (*n* = 30, 71%) and the remaining participants expressed a preference for face-to-face only (*n* = 12, 29%).

**Fig. 4:**
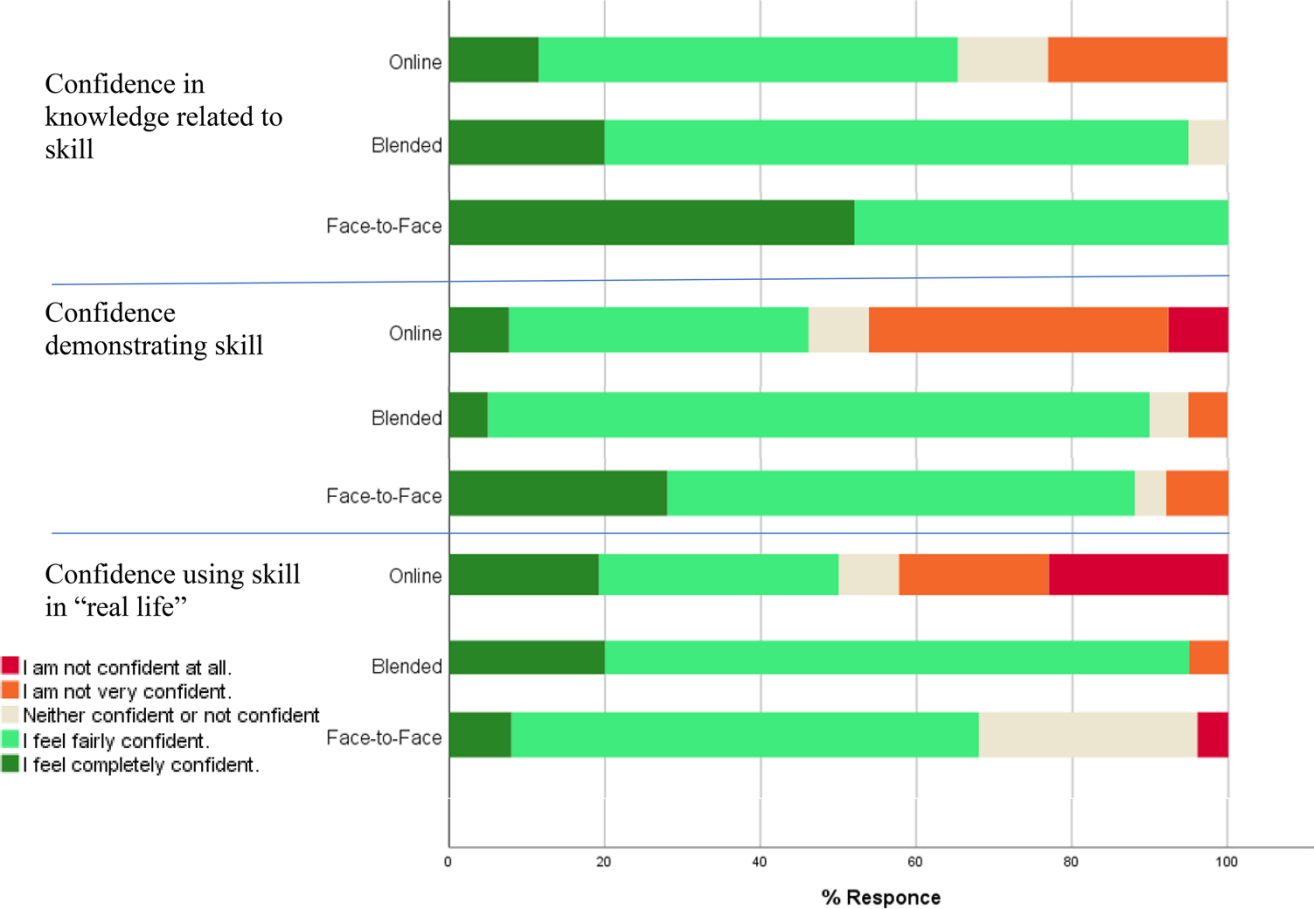
Response by group on “confidence in your knowledge of the skill learnt”

Analysing both skills together, when asked about their perceived confidence in knowledge of the skill, demonstrating the skill and confidence related to performing the skill in a clinical situation, the face-to-face and blended groups consistently reported higher levels of confidence than the online group (see Fig. 4). For all three questions, there was a statistically significant difference between learning groups, indicating a relationship between learning group and perceived self-efficacy in skill knowledge and performance (Fisher’s exact test score 19.6, *p* = 0.001 for knowledge confidence; 19.13, *p* = 0.003 for confidence in demonstrating the skill; 23.9, *p* ≤ 0.001 for perceived confidence performing the skill in “real life”).

**Fig. 5:**
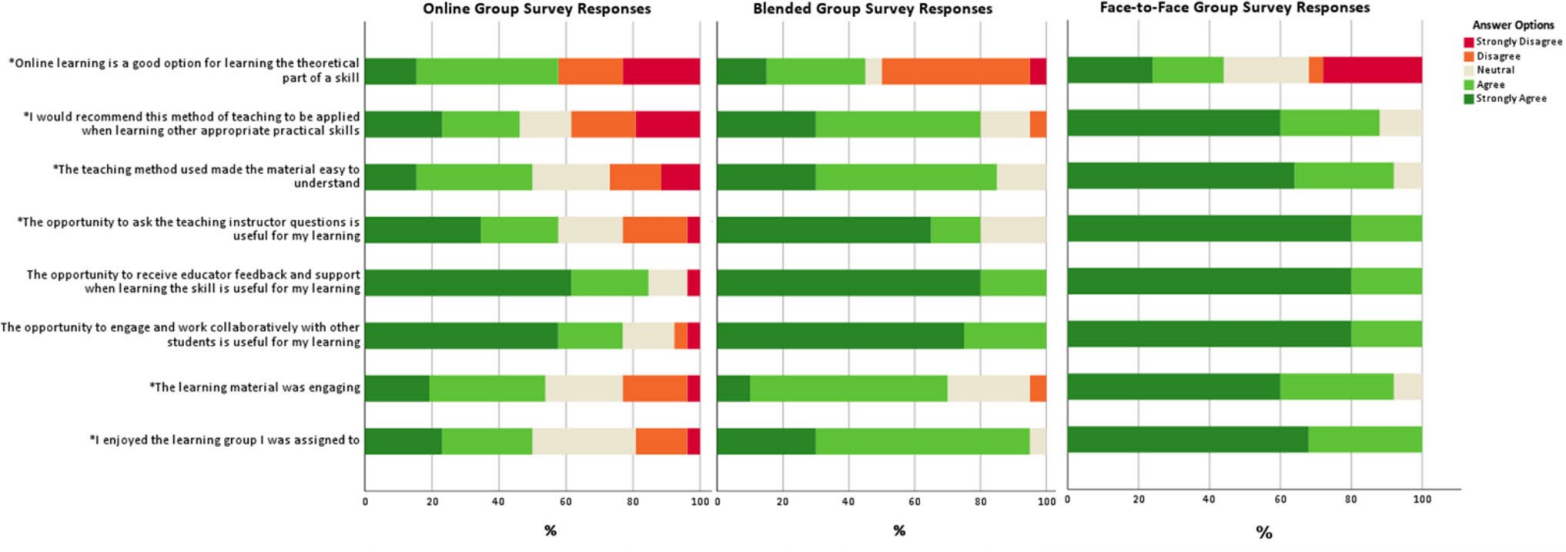
Survey responses across learning groups. *indicates a statistically significant difference between teaching groups

Student experience of the learning material clearly differed between teaching approaches with a statistically significant between-group difference observed across many survey responses. In general, those in the fully online group had a more negative experience. There was a statistically significant difference between teaching approaches in subjective enjoyment of the learning experience (*p* < 0.001), how engaging the material was perceived to be (*p* = 0.002), ease of understanding (*p* = 0.002), perceived use of the opportunity to ask a question of an instructor (*p* = 0.003), whether participants would recommend the teaching approach they were exposed to (*p* = 0.006) and whether they thought that the theoretical parts of a skill would be suited to online teaching methods (*p* = 0.006) (see Fig. 5).

## Discussion

This research aimed to assess the impact of three teaching approaches on practical skill acquisition, namely, if face-to-face, blended or online teaching were equally effective for building student competency and confidence in practical skills requiring auditory training and haptic awareness. Participant satisfaction with each teaching method was also evaluated. Determining whether there is a difference between teaching methods for practical skill acquisition could inform the future of digital health education moving forwards.

Students in the face-to-face and blended learning groups reported greater satisfaction with their assigned learning group, demonstrating greater confidence in their skill knowledge and performance compared to the online learning groups. Previous literature exploring digital and online resource use in medical education have concluded that blended learning is appropriate for theoretical learning; our study further advances knowledge on digitally enhanced practical teaching as it supports blended teaching for the acquisition of skills requiring auditory and tactile training. Our results also indicate that fully online learning is not as effective for the acquisition of such skills. These results were consistent across both gross and sub-competency skill acquisition, indicating that the overall teaching method is a major variable and is likely to be independent of the teaching material.

When sub-competencies were examined across both skills, the online learning group performed poorly on aspects of the taught skill which required haptic awareness (e.g. cuff placement), as well as on technical aspects of the skill that did not require sensory feedback (e.g. site rotation). For example, the online learning group performed poorly at cuff placement during demonstration of BPM, which is a sub-competency requiring visual and palpation skills, the latter of which would not be easily acquired in a fully online format without practice. Additionally, the online learning group performed poorly on technical sub-competencies of SKM, such as knowing that measurement sites need to be rotated and that a minimum of two measurements are required per site. Importantly, these sub-competencies are knowledge based and do not require sensory training. These results lead us to believe that rather than considering what skills are best suited to a given teaching method, the more pertinent point may be to consider the quantity of time spent learning online or the proportion of online learning within a blended format. Although skills requiring certain senses may appear better suited to a given teaching method, the acquisition of both knowledge and skill can suffer if teaching is fully online, which may be attributed to a lack of motivation, poor engagement and reduced feedback [27]. The educational setting itself could also explain some of the between-group differences reported, with a clinical setting leading to productive levels of stress which may benefit learning experience [28].

These considerations could be important in informing the design and development of blended learning materials. Our work could help to inform decisions on pedagogical pathways and educational strategies, as health science education continues to adapt to changing times with pressure on resources such as space and faculty time [29, 30]. Our work has shown that students appear to have the greatest success in all components of a skill (i.e. knowledge as well as competencies) when a blended or fully face-to-face learning approach is offered; however, the overall preference from students is for a blended approach to practical skill teaching, which is similar to previous literature in the area [3, 6, 9, 11–13]. This work has already informed a medical school online education strategy and an open educational resource which is available at https://hub.teachingandlearning.ie/resource/depth-digitally-enhanced-practical-teaching-in-the-health-sciences/ and https://tcdmedonline.ie/DEPTH/story.html. Further studies might benefit from deciphering how best to deliver blended learning, for example, giving students the opportunity to choose whether to engage with a proportion of their teaching material online or in person, thereby increasing student control over their studies while maintaining some in-person classes.

The literature on blended learning highlights the benefits of having access to learning materials prior to in-person skill sessions [5]. Receiving expert feedback during in-person practice then helps to consolidate theoretical learning and support students in acquiring the technical skills necessary to perform a skill successfully [5, 9]. In the current study, students allocated to the online learning group choosing not to interact with the online discussion forum raises concerns around the efficacy of online forums in building skill knowledge and highlights the need for real-time feedback when practicing clinical skills. While these findings contrast with those of a meta-analysis which concluded that online learning was as effective as traditional in-person teaching methods [31], there appears to be mounting evidence in the area of practical skill acquisition that a blended approach is preferable and effective. Studies included in the aforementioned meta-analysis [31] reported primarily on knowledge acquisition rather than skill acquisition and teaching methods were assessed mostly through multiple choice questions or tests rather than an examination of a practical competency.

While survey results indicated that those in the fully online group had a more negative learning experience than those in the other two groups, it is worth noting that their experience was not entirely negative, and results may seem less favourable towards a fully online teaching method due to the almost exclusively positive feedback from those in the face-to-face and blended learning groups. For example, no participant in the face-to-face or blended groups disagreed with the statement that the learning method was enjoyable or disagreed with the statement that the learning method helped them to understand the material. This is in comparison to 19% (who disagreed with the statement that the learning material was enjoyable) and 27% (who disagreed with the statement that the learning method helped them to understand the material) in the online group which, although considerably higher than 0% is still a minority of that group (Fig. 5). The results of this work show a divide in the student community, with a tendency for slightly more than half of the participants in the online group to respond positively to statements about the teaching method and the remainder responding either neutrally or negatively. This divide in opinion was not evident in the other two learning groups. Further studies may investigate the reason for the differences in opinions seen in the online groups and critically, whether those who enjoy online learning are more effective online learners.

### Strengths and Limitations

A strength of this study is its design which ensured that where possible learning materials were appropriate for the teaching method but the same between groups (e.g. a video demonstrating a skill was provided for online learners and the same person demonstrated the skill in the same way in person for those in the face to face group). This design was used to try to ensure that the main variable between groups was the teaching method and not the quality of the teaching material provided. Although there are many other factors that can affect skill acquisition such as learning style and personality [32, 33], the random allocation used in this study would help ensure confounding factors did not affect results. We can have confidence in the results seen since they are consistent between skills examined (BPM and SKM) and between objective and subjective measures (competence and survey results). Another strength is that one competency assessor was blinded to participant group allocation, which helped ensure objectivity in scoring competencies. Finally, our competency results were complemented by participant self-reported survey results, which gives our data an additional dimension, ensuring student perspectives are included.

There are some limitations to our work as well. The fact that teaching in this study was conducted outside of participant course requirements and was therefore not for credit may have negatively affected participants’ motivation to learn. However, as participation was voluntary, it is reasonable to assume a certain level of motivation. We did not measure whether students assigned to the blended or online learning groups engaged with their online learning materials, and the impact of adherence on outcomes. A lack of student engagement with online learning materials has been identified as a major barrier to practical skill teaching and can be explained by low motivation, reduced attention and poor time management (7). We did not measure whether certain components of the blended teaching material led to greater levels of competence than others. It could be argued that it was mainly the face-to-face aspects of the blended learning experience which resulted in higher competence; however, we were not in a position to determine this since each teaching method was developed as a whole and individual components were not designed to be isolated.

Another limitation is the possibility of cross-contamination. Students were encouraged to practice the skills prior to their in-person demonstrations. Although students were randomised into their learning group, they were all recruited from TCD’s School of Medicine and it is therefore possible that friends in different learning groups may have practiced together. Additionally, as all participants were undergraduate students, results are therefore not transferable to a postgraduate cohort.

It is worth noting that for BPM, overall skill competency across all three learning groups was below 50%. Research has shown that the noise of a treadmill or bike, and moving muscles and joints, can interfere with the Korotkoff sounds (the sound listened to during auscultation of the brachial artery when taking blood pressure measurement), making them difficult to distinguish and accurately measure (27, 31). It is therefore possible that each learning group found manually measuring blood pressure during exercise a particularly difficult skill to learn. Providing students with more time to practice the skill and familiarising themselves with the interfering external noise of the exercise bike may have enhanced student success. While students were encouraged to practice the skill at home, they were unlikely to have had a cycle ergometer to hand. Blood pressure measurement during exercise may be a particularly difficult skill to learn and may require additional in-person practice, regardless of teaching approach, to develop the auditory awareness necessary for an accurate measurement.

## Conclusions

Results of this work revealed that a fully online teaching method continuously led to poorer outcomes than both blended and face-to-face teaching methods. Our results indicate that the opportunity to ask a question of an academic is not considered to be as useful if online; furthermore, material is not considered to be as engaging or enjoyable and is considered harder to understand when presented online when compared to a blended or face-to-face delivery. Those who engaged with learning material fully online were also less confident in both the knowledge related to the skill they learnt and completing the skill itself. Survey findings revealed that participants in the online learning group found learning materials less engaging and found that the teaching method negatively impacted their ability to understand the learning materials when compared with the other teaching methods. The reason for this could be that although encouraged to practice on volunteers, the online learning group may have been disadvantaged in their exposure to material via two senses (i.e. sight/sound) when engaging with the learning resources, as opposed to the blended and face-to-face learning groups who could benefit from tactile training in gathering skin folds and applying cuffs during in-person practice sessions [21]. It is likely that the lack of in-person practice with an academic and lack of real-time feedback on their technique disadvantaged the online learning group, resulting in poorer competency levels.

Overall, these findings indicate that while online teaching supported some students in achieving practical skill competency, blended teaching approaches are both more effective and preferred by students for skills requiring tactile and auditory training. This study advances health science education by demonstrating the suitability and acceptability of blended learning for skills not traditionally deemed suited to digitally enhanced teaching methods.

## Data Availability

Data and other relevant materials related to this work can be accessed via the Open Science Framework; DOI 10.17605/OSF.IO/DZG6T link; 10.17605/osf.io/dzg6t.
